# Validation and discussion of clinical practicability of the 2022 graded prognostic assessment for NSCLC adenocarcinoma patients with brain metastases in a routine clinical cohort

**DOI:** 10.3389/fonc.2023.1042548

**Published:** 2023-03-20

**Authors:** C. Schröder, P. Windisch, J. Lütscher, D. R. Zwahlen, R. Förster

**Affiliations:** Clinic for Radiation Oncology, Cantonal Hospital Winterthur, Winterthur, Switzerland

**Keywords:** adenocarcinoma, brain metastases, overall survival, prognosis, lung

## Abstract

**Introduction:**

The goal of this analysis is to validate the 2022 graded prognostic assessment (GPA) for patients with brain metastases from adenocarcinoma of the lung and to discuss its clinical practicability.

**Methods/material:**

137 patients with adenocarcinoma of the lung were included in this analysis. The disease specific GPA for NSCLC, Lung-molGPA and the GPA for NSCLC adenocarcinoma were calculated. Overall survival was calculated for each GPA group. Additionally, expected and actual OS in the prognostic groups of the GPA available at the time of the patients’ diagnosis was compared.

**Results:**

Median overall survival (OS) from diagnosis of brain metastases was 15 months (95% confidence interval (CI) 9.7–20.3 months). The median OS in the three individual prognostic groups was 7 months for GPA 0-1, 16 months for GPA 1.5-2, 33 months for GPA 2.5-3 and not reached for GPA 3.5-4 (p<0.001). Median survival times for the individual groups were similar to those published in the original GPA publication. Regarding the expected and actual OS when using the available GPA at the time of diagnosis there was an underestimation of survival of more than 3 months for all except the worst prognosis group.

**Conclusion:**

We were able to validate the 2022 GPA for NSCLC adenocarcinoma patients with brain metastases in a similar cohort from a non-academic center. However, the practical applicability regarding the expected median OS might be limited due to the constantly evolving treatment landscape and the consecutive improvement in overall survival.

## 1 Introduction

Brain metastases are quite common in patients with lung cancer with up to 50% of patients either presenting with or developing brain metastases during the course of the disease (1–3). With lung cancer being the second most common cancer and the most common cause of death from cancer worldwide, this results in a substantial amount of patients (4).

The diagnosis of brain metastases leads to complex problems in the management of these patients. On the side of symptom burden, brain metastases may lead to various, possibly severe symptoms, i.e. neurocognitive impairment, personality changes, fluctuating vigilance, epileptic seizures and loss of mobility (5). Regarding therapeutic options, limited penetration of systemic drugs, the loss of targetable mutations and the selection of resistant clones during long disease courses may lead to significant therapeutic challenges (6).

Depending on several factors, i.e. targetable mutations or number of brain metastases, there are multiple treatment options for brain metastases, including radiotherapy (stereotactic or whole brain radiotherapy), surgery and systemic therapy penetrating the blood-brain barrier (chemotherapy, immunotherapy or targeted therapy) (7).

Prognostic scores can be helpful to guide treatment for the primary, for the extracranial metastatic disease, and for the brain metastases. Due to improved therapeutic options, the life expectancy of patients with brain metastases has improved over the last decades (8–10). Consequently, prognostic factors, i.e. the recursive partitioning analysis (RPA) and the graded prognostic assessment (GPA), had to be adapted over time (6, 11–15). Sperduto et al. published the latest version of the GPA for patients with lung cancer in 2022 including the Karnofsky performance status (KPS), age, number of brain metastases, the absence of extracranial metastases, the mutation status (EGFR, ALK) and PD-L1 status (6).

The basis for the development of the GPA scores was an academic cohort. Therefore, the primary aim of this analysis was to validate the new GPA for lung adenocarcinoma with the data from our non-academic tertiary care hospital. Secondarily, we aimed to assess whether the GPA that was available at the time of the patients’ diagnosis would have accurately estimated the overall survival.

## 2 Materials and methods

### 2.1 Patient and treatment related characteristics

A single institutional database from a certified lung cancer unit at a cancer center was searched for patients with adenocarcinoma of the lung with a diagnosis of brain metastases from January 2015 – December 2020. 166 patients were initially identified. Of those, 29 patients had to be excluded from this analysis in accordance with the exclusion criteria stated by Sperduto et al. (6). In ten cases, patient had leptomeningeal disease, 18 patients received best supportive care (BSC) and in one case the amount of available data was insufficient.

Median age of patients at the diagnosis of brain metastases was 67 years (range, 42 – 87 years) with a female predominance (n = 76; 55.5%). The majority of patients (n = 106; 77.4%) presented with metastatic disease upon first diagnosis. Most patients (n = 76; 55.5%) received systemic therapy as initial treatment for the primary tumor and radiotherapy as primary treatment for the brain metastases (n = 97; 70.8%). The most common type of cranial radiotherapy used in this cohort was whole brain radiotherapy (WBRT, n = 61, 44.5%) followed by stereotactic radiotherapy (SRT, n = 52, 38%) and partial brain radiotherapy (n = 18, 13.1%). In total, 47 patients (34.4%) had an intracranial relapse. The intracranial relapse rates for patients receiving SRT and partial brain radiotherapy were very similar (52% vs. 55%) and lower with WBRT (16.4%). Patients that initially received WBRT had the following treatment in case of cranial relapse: best supportive care (BSC) (n = 2, 20%), surgical resection in (n = 2, 20%), systemic therapy in (n = 3, 30%), re-irradiation in (n = 3, 30% with 2 patients SRT, 1 patient re-WBRT). Patients with initial partial brain RT received surgical resection (n = 1, 10%), systemic therapy (n = 4, 40%) or re-irradiation (n = 5, 50% with 1 patient WBRT, 1 patient partial brain, 3 patients SRT). In patients with initial SRT, the most common therapy in case of recurrence was re-irradiation (n = 16, 59.2% with 8 patients WBRT, 8 patients SRT), followed by systemic therapy (n = 7, 25.9%), surgical resection (n = 3, 11.1%) and BSC (n = 1, 3.7%). Additional patient and treatment related characteristics are shown in **Table 1**.

**Table 1:** Patient characteristics.

		n	%
Sex	male	61	44.5
	female	76	55.5
Initial T stage	1	20	14.6
	2	29	21.2
	3	29	21.2
	4	45	32.8
	x	14	10.2
Initial N stage	0	21	15.3
	1	20	14.6
	2	43	31.4
	3	44	32.1
	x	9	6.6
Initial M stage	0	31	22.6
	1	106	77.4
PD-L1 (analog (6))	negative	32	23.4
	positive	54	39.4
	unknown	51	37.2
ALK	negative	92	67.2
	positive	6	4.4
	unknown	39	28.5
EGFR	negative	88	64.2
	positive	19	13.9
	unknown	30	21.9
KRAS	negative	75	54.7
	positive	37	27.0
	unknown	25	18.2
Number of brain metastases	1	51	37.2
	2-4	27	19.7
	>=5	59	43.1
Extracranial metastases	not present	56	40.9
	present	81	59.1
Status primary	not controlled	82	59.9
	controlled	55	40.1
Status extracranial metastases	not controlled	61	44.5
	controlled	76	55.5
KPS	90-100%	21	15.3
	80%	66	48.2
	<=70%	50	36.5
Initial treatment for primary	none	11	8.0
	systemic therapy only	76	55.5
	radiotherapy only^*^	7	5.1
	radiotherapy only^+^	2	1.5
	resection only	5	3.6
	multimodal: resection + systemic therapy^#^	14	10.2
	multimodal: radiotherapy^*^ + systemic therapy^¥^	10	7.3
	multimodal: radiotherapy^+^ + systemic therapy^¥^	2	1.5
	multimodal: resection + radiotherapy + systemic therapy^#^	10	7.3
Initial therapy brain metastases	resection	25	18.2
	radiotherapy	97	70.8
	systemic therapy	15	10.9
Type of cranial radiotherapy	WBRT	61	44.5
	partial brain radiotherapy	18	13.1
	SRT	52	38.0
Total		137	100.0

* Curative intent; + Palliative intent; # neoadjuvant or adjuvant; ¥ sequential or concomitant.

### 2.2 Endpoints and statistical analysis

The disease specific GPA for NSCLC, Lung-molGPA and the GPA for NSCLC adenocarcinoma were calculated according to the published criteria (6, 14, 15). Survival was calculated from the date of diagnosis of disease as well as the diagnosis of brain metastases until the date of death or last follow-up. Overall survival (OS) was calculated using the Kaplan Meier method. Median OS for the individual GPA prognostic groups was calculated. For group comparison, the log-rank test was used. Due to the very limited number of patients in the prognostic group with GPA 3.5-4.0, this group was excluded from the log-rank test. A p-value ≤ 0.05 was considered statistically significant. For statistical analysis, SPSS version 28 (Statistical Package for Social Sciences, IBM Corp., Armonk, NY, USA) was used. This analysis was approved by the responsible ethics committee.

## 3 Results

### 3.1 Validation of the 2022 NSCLC adenocarcinoma GPA

**Figure 1:**
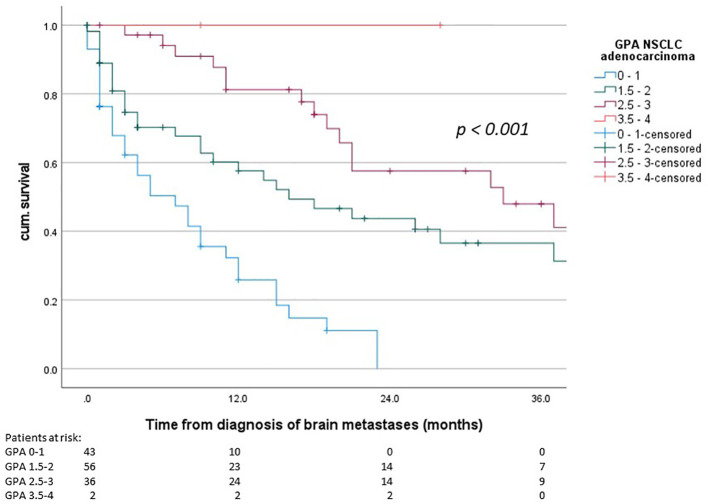
Overall survival from diagnosis of brain metastases, GPA groups according to the 2022 GPA NSCLC adenocarcinoma (p < 0.001).

Median overall survival from first diagnosis of disease was 24 months (95% confidence interval (CI) 16.1 – 32.0 months) and median survival from the diagnosis of brain metastases was 15 months (95% CI 9.7 – 20.3 months). The median OS in the three individual prognostic groups was 7 months (GPA 0-1), 16 months (GPA 1.5-2) and 33 months (GPA 2.5-3), respectively (Chi-Square 34.013, p < 0.001). For the best prognostic group (GPA 3.5 – 4) the median OS was not reached since more than half of the patients were still living. The survival curves are shown in **Figure 1**. The comparison of the distribution of patients and the median OS in our cohort and the original cohort [from (6)] is shown in **Table 2**.

**Table 2:** Comparison of the distribution of patients and median OS of the original cohort and our cohort.

	Distribution of patients N (%)		Median overall survival MS, mo (interquartile range)	
GPA scores	Sperduto et al. (6)	Our cohort	Sperduto et al. (6)	Our cohort
0 - 1	622 (21.8)	43 (31.4)	6 (2, 13)	7 (2, 15)
1.5 - 2	1177 (41.2)	56 (40.9)	15 (5, 38)	16 (3, 51)
2.5 - 3	847 (29.7)	36 (26.3)	30 (12, NR)	33 (18, 46)
3.5 - 4	210 (7.4)	2 (1.5)	52 (25, 69)	NR (NR, NR)
Overall	2856 (100)	137 (100)	17	15

NR, not reached.

### 3.2 Application of GPA to predict survival in clinical practice

In this cohort, 45 patients had the diagnosis of brain metastases before the publication of the Lung-mol GPA in 06/2017. For these patients (diagnosed 01/2015 – 05/2017) the disease specific GPA from 2012 would have been applied (14) in clinical practice. For the patients diagnosed from 06/2017 – 12/2020 the Lung-molGPA would have been applied (15).

The median overall survival of the patients diagnosed until the end of 05/2017 was 18 months and 14 months for the patients diagnosed thereafter (06/2017 – 12/2020). The comparison of the expected median OS according to the respective GPA in the different prognostic groups and the actual median OS in this cohort is shown in **Table 3**. Notably, the separation between survival curves is still statistically significant for both groups (until end of 05/2017 p = 0.035, from 06/2017 p < 0.001).

**Table 3:** Comparison of the expected median OS according to the available prognostic score and the actual median OS of patients treated in the time period.

	Score available during patient treatment period			
	until end of 05/2017: disease specific GPA (14)		from 06/2017: Lung-molGPA (adenocarcinoma) (15)	
GPA scores	Original data (14) Median survial, mo	Our cohort (n = 45) Median survial, (interquartile range)	Original data (15) Median survial, mo	Our cohort (n = 92) Median survial, mo (interquartile range)
0.0-1.0	3.0	4 (1, 23)	6.9	7 (2, 12)
1.5-2.0	5.5	26 (15, 67) *	13.7	19 (7, 32) *
2.5-3.0	9.4	15 (6, 18) *	26.5	37 (12, NR) *
3.5-4.0	14.0	–	46.8	NR (NR, NR)
Overall	7.0	18	15.2	14

* Difference original data vs. our cohort > 3 month; NR, not reached.

## 4 Discussion

With the constant development of new therapies for non-small cell lung cancer (NSCLC) and as a consequence, the improved prognosis of patients, there also has been an adaptation of the proposed prognostic scores. The Radiation Therapy Oncology Group (RTOG) recursive partitioning analysis (RPA) for all patients with brain metastases was introduced in 1997 (11, 12). It included age, KPS score, controlled primary tumor and the absence of extracranial metastases. In 2008, a graded prognostic assessment (GPA) for patients with brain metastases was introduced by Sperduto et al. including similar factors like the RPA (13). The same group proposed a disease specific GPA (with NSCLC and small cell lung cancer (SCLC) having a similar scoring system but a different prognosis) 4 years later in 2012; with again similar factors in the lung cancer group (age, KPS score, absence of extracranial metastases, number of brain metastases) (14). With further improvement of therapies and subsequently survival, Sperduto et al. further adapted their prognostic score and introduced the Lung-molGPA in 2017, including molecular markers, i.e. EGFR and ALK, for the first time (15). This score was recently further updated with a newly introduced separation of NSCLC adenocarcinoma and NSCLC non-adenocarcinoma, not only regarding the prognosis as in the the Lung-molGPA but also for the score itself (6). Additionally, the presence of a PD-L1 status was included. Furthermore, during the adaption of the scores the cutoff for age was raised from 60 to 70 years (from 2017) and the grading for the number of brain metastases was changed (also from 2017) (14, 15).

Regarding survival, the median overall survival more than doubled from the publication of the RPA (7 months) to the latest 2022 GPA (17 months) (6, 11, 12). In our cohort, the median overall survival was inferior with 15 months, which rather resembles the 2017 Lung-molGPA adenocarcinoma cohort (15). However, the difference was very small with 2 months and likely attributable to the distribution of included patients in the prognostic groups. In our cohort, 72.3% of patients were in the worst two prognostic groups (GPA 0-1, GPA 1.5-2) as opposed to 63.0% in the 2022 cohort of Sperduto et al. (6). Generally, the median OS in the individual subgroups of the original paper and this cohort were very similar with a maximum difference of three months in the GPA 2.5 – 3 group. For the best prognostic group (GPA 3.5 – 4.0), no conclusion is possible due to the very limited amount of patients in cohort analyzed in this work. The median overall survival in the individual prognostic group was very similar with the 2022 GPA being highly significant for overall survival (p < 0.001). This prognostic group was also rather underrepresented in the original cohort (7.4%) and even lower in this cohort (1.5%). This might be attributable to this being a non-academic cohort.

Although prognostic scores can be a helpful tool in clinical practice to estimate prognosis and guide treatment decisions, there are some critical aspects to consider. In case of the 2022 GPA, this is for once the more detailed separation of the KPS (e.g. ≤ 70%, 80%, 90-100% for adenocarcinoma and ≤ 60%, 70%, 80%, 90%, 100% for SCLC) which might be challenging to obtain correctly in clinical practice. There are known biases for the estimation of the performance status due to the subjective nature and KPS may vary in these patients on a daily basis (16–20). Another factor to consider is the reason for the constant updates of the GPA as a consequence of the constant improvement of therapeutic options and hence, the overall survival of these patients. This might be less important for some tumor entities but especially in lung cancer there is rapid development, e.g. regarding CNS penetrating systemic therapies (21–26). In this scenario, a score that is based on an older cohort of patients might not be entirely accurate even at the time of publication. This cohort for example was treated between January 2015 and December 2020. The 2017 Lung-mol GPA [cohort 2006 – 2014 (15)] was published in June 2017 and would have been used for most patients in this cohort (92 patients (67%) were diagnosed from June 2017 on). Although the separation of the survival curves with the 2017 Lung-molGPA was still very good in the cohort analyzed in this work, the median survival times differed significantly for some groups. For the prognostic group with a GPA 0-1, the median OS was quite similar (6.9 months in the work of Sperduto et al. (15) vs. 5 months in this cohort), but for the GPA scores 1.5-2 (13.7 months in (15) vs. 21 months in this cohort) and 2.5-3 (26.5 months in (15) vs. 37 months in this cohort) the median OS differed by up to 10 months. Therefore, prognostic scores should be used with caution, especially in rapidly evolving fields.

The limitations of this analysis lie in the limited amount of patients, specifically across subgroups, and the retrospective nature of the analysis.

## 5 Conclusion

In conclusion, the 2022 GPA for NSCLC adenocarcinoma patients with brain metastases was validated in a similar cohort from a non-academic center. However, the expected median OS might likely change dynamically in this patient group due to the rapidly evolving therapeutic options and the subsequently constantly improving OS.

## Data availability statement

The data analyzed in this study is subject to the following licenses/restrictions: Hospital internal database. Requests to access these datasets should be directed to christina.schroeder@ksw.ch.

## Ethics statement

The studies involving human participants were reviewed and approved by Cantonal Ethics Committee Zurich (BASEC-Nr. 2020-02112, 2020-02124). The patients/participants provided their written informed consent to participate in this study. Consent for patients who were deceased was covered BASEC Nr. 2020-02124, which is an Ethics approval according to article 34 HFG.

## Author contributions

All authors contributed to the article and have read and agreed to the published version of the manuscript.
